# Lead time on confirmatory test after abnormal Pap test in the COVID-19 era

**DOI:** 10.1097/MD.0000000000027327

**Published:** 2021-10-01

**Authors:** Miseon Kim, Nara Lee, Seyeon Won, Ju-Hyun Kim, Mi Kyoung Kim, Mi-La Kim, Yong Wook Jung, Bo Seong Yun, Seok Ju Seong

**Affiliations:** a Department of Obstetrics and Gynecology, CHA Gangnam Medical Center, CHA University School of Medicine, Seoul; b Department of Obstetrics and Gynecology, CHA Ilsan Medical Center, CHA University School of Medicine, Goyang, Korea.

**Keywords:** cervical neoplasm, colposcopy, COVID-19, Papanicolaou test

## Abstract

During the COVID-19 pandemic, there are concerns about medical delay, including confirmatory tests after screening for various cancers. We evaluated the lead time to a confirmatory test after an abnormal screening Papanicolaou (Pap) test in women before the COVID-19 period and during the COVID-19 period.

The medical records of 1144 women who underwent colposcopy at a single institution located in Seoul after abnormal Pap results from January 2019 to December 2020 were reviewed. The lead time to colposcopy from the Pap test between 2019 and 2020 was compared; the adverse factors for a long lead time to colposcopy were also evaluated.

Age, residence, institution, and the Pap results did not differ between women who underwent colposcopy in 2019 (n = 621) and 2020 (n = 523). The time to colposcopy from the Pap test was also not different. A higher number of women were diagnosed with high-grade dysplasia in 2020 and underwent excision procedures; however, the difference was not statistically significant. Instead, patients’ residence, institution of the Pap test, and results of the Pap test were associated with a long lead time to colposcopy of >6 weeks.

The lead time to colposcopy from the abnormal Pap test was not delayed in the COVID-19 era compared to before. However, regional factors could affect a long lead time.

## 1 Introduction

The Papanicolaou (Pap) test was first introduced as a method of cytology-based screening for cervical cancer in 1941; subsequently, national screening programs using the Pap test with 3- to 5-year intervals have been the most common strategy in several countries.^[1,2]^ Despite this effective screening, approximately 570,000 new cases of cervical cancer and 311,000 related deaths were reported in 2018.^[3]^ The steady cervical cancer mortality may result from a lack of screening Pap tests or a failure to detect cell abnormalities in the Pap test. In addition, a lack of immediate follow-up tests after an abnormal Pap test must also be considered. The American Society for Colposcopy and Cervical Pathology released evidence-based consensus guidelines for the adequate management of abnormal Pap results.^[4]^ Colposcopy is considered to be the primary diagnostic approach to confirm the abnormal Pap results; additionally, an immediate colposcopy is recommended as a follow-up to Pap test results of atypical squamous cells of undetermined significance (ASCUS) with high-risk human papillomavirus (HPV) and low-grade squamous intraepithelial lesions (LSIL); atypical squamous cells cannot exclude high-grade squamous intraepithelial lesions (ASC-H), and high-grade squamous intraepithelial lesions (HSIL).

The recommendations regarding the time to the confirmatory test exist in several countries based on expert consensus; however, they are significantly heterogeneous, although high-grade cytological abnormalities commonly have shorter follow-up intervals than low-grade cytological abnormalities.^[5]^ The present strategy is to perform colposcopy within 30 days for women with suspected invasive cancer, within 60 days for women with high-grade cytological abnormalities, and within 90 days for women with low-grade cytological abnormalities, such as ASCUS with high-risk HPV and LSIL.^[6]^ However, it is known that <75% of women receive follow-up testing after abnormal Pap results.^[7]^ In addition, the 2019 coronavirus disease (COVID-19) pandemic declared by the World Health Organization in March 2020 has impacted the diagnostic procedures in various medical fields, including those used for cancer prevention. Several countries have disturbed cancer screening activities during the COVID-19 pandemic, including the Pap test for cervical cancer screening. Such changes have the potential to delay cancer detection and increase the proportion of patients with advanced-stage disease, which can result in an increased overall incidence and mortality of cervical cancer.

In this study, we aimed to investigate whether the time to colposcopy as a confirmatory test after the abnormal Pap test was delayed in the COVID-19 era. Thus, we compared the lead time after an abnormal screening Pap test in women before the COVID-19 era (January 2019–December 2019) and during the COVID-19 era (January 2020–December 2020). By analyzing the clinical information of various patients, we also evaluated the independent factors that result in delayed colposcopy after the abnormal Pap test.

## 2 Materials and methods

### 2.1 Patients

The study cohort was retrospectively recruited. The study protocol was revised and approved by institutional review board, and research was conducted in accordance with the 1964 Declaration of Helsinki and its later amendments. The medical records of 1144 women with abnormal Pap results who underwent colposcopy with punch biopsy at a single institution located in Seoul, Korea, from January 2019 to December 2020 were reviewed. The exclusion criteria were as follows: women who had been diagnosed with cervical dysplasia or cancer previously, women who underwent Loop electrosurgical excision procedure or laser therapy on the cervix, women who underwent the Pap test and colposcopy at the same time; women who underwent hysterectomy; and women without the exact data for the Pap test.

### 2.2 Variables

The Pap test results were classified according to the pathological findings using the 2001 Bethesda system. The abnormal Pap test results included ASCUS, atypical glandular cells, ASC-H, LSIL, HSIL, squamous cell carcinoma (SCC), adenocarcinoma in situ, or adenocarcinoma. The biopsy results were classified as normal, cervical intraepithelial neoplasia (CIN)1, CIN2, CIN3/carcinoma in situ (CIS), and invasive cancer. The lead time to colposcopy from the Pap test between 2019 and 2020 was compared, and factors associated with a long lead time of >6 weeks to colposcopy were also evaluated.

### 2.3 Statistical analysis

Student *t* test and the *χ*^2^ test were used to evaluate continuous and categorical variables, respectively. The Mann–Whitney *U* test and Fisher exact tests were used for the corresponding nonparametric statistics. Logistic regression was used to identify the independent factors associated with a long lead time to colposcopy using the Pap test. Variables with *P* ≤ .25 were included in the multivariate analysis, and *P* < .05 was considered to be significant. IBM SPSS Statistics for Windows, version 24.0 (IBM Corp., Armonk, NY) was used for statistical analyses.

## 3 Results

### 3.1 Baseline characteristics

The baseline characteristics are presented in Table 1. Among 1144 women, 621 women underwent the Pap test in 2019 and 523 women underwent the Pap test in 2020. The proportion of women who visited the institution for colposcopy decreased to 84.2% in 2020 compared to 2019. Age (years; 37.5 ± 10.4 vs 38.3 ± 10.9, *P* = .228), patients’ residence (Seoul: 72.6% vs 75.2%, *P* = .199), institution where the Pap test was performed (same as those of colposcopy; 53.6% vs 54.2%, *P* = .846), and results of the Pap test (ASCUS: 56.0% vs 55.7%, *P* = .525) were not different between women who underwent colposcopy in 2019 and 2020.

**Table 1:** Baseline characteristics.

	Before the COVID-19 era (2019, n = 621)	In the COVID-19 era (2020, n = 523)	*P*
Age	37.5 ± 10.4	38.3 ± 10.9	.228
Residence			.199
Seoul	451 (72.6)	394 (75.2)	
Metropolitan around Seoul	118 (19.0)	97 (18.5)	
Rural area	52 (8.4)	33 (6.3)	
Institution of the Pap			.846
Same institution	333 (53.6)	284 (54.2)	
Others	228 (46.4)	240 (45.8)	
Results of the Pap			.525
ASCUS	348 (56.0)	292 (55.7)	
LSIL	141 (22.7)	123 (23.5)	
ASC-H	47 (7.6)	53 (10.1)	
HSIL	71 (11.4)	48 (9.2)	
AGC	11 (1.8)	7 (1.3)	
SCC	3 (0.5)	1 (0.2)	
Time to colposcopy from the Pap	27.9 ± 10.1	27.2 ± 18.1	.576

AGC = atypical glandular cells, ASC-H = atypical squamous cells cannot exclude high-grade squamous intraepithelial lesions, ASUCS = atypical squamous cells of undetermined significance, COVID-19 = Coronavirus disease, HSIL = high-grade squamous intraepithelial lesions, LSIL = low-grade squamous intraepithelial lesions, Pap = Papanicolaou test, SCC = Squamous cell carcinoma.

### 3.2 Time to colposcopy from the Pap test

**Figure 1:**
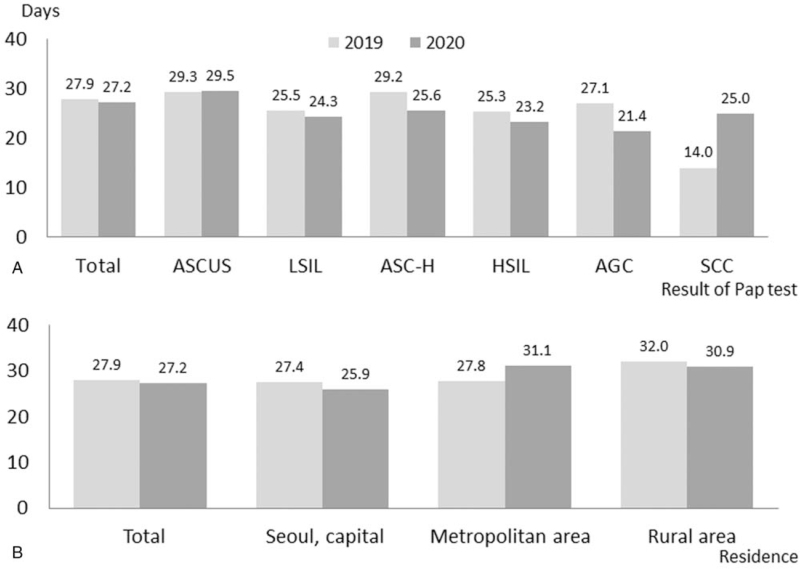
Comparison of lead times to colposcopy from the Pap test for screening between 2019 and 2020 (A) by the results of the Pap test and (B) residences of the patients. AGC = atypical glandular cells, ASC-H = atypical squamous cells cannot exclude high-grade squamous intraepithelial lesions, ASUCS = atypical squamous cells of undetermined significance, HSIL = high-grade squamous intraepithelial lesions, LSIL = low-grade squamous intraepithelial lesions, SCC = squamous cell carcinoma.

The time to colposcopy from the Pap test was shorter in women with SCC results and longer in women with ASCUS results (days; ASCUS = 29.4, LSIL = 24.9, ASC-H = 27.3, HSIL = 24.5, atypical glandular cells = 24.9, and SCC = 16.8). The lead time was shorter in women living in Seoul and longer in women living in rural areas (days; Seoul = 26.7, metropolitan areas around Seoul = 29.2, and rural areas = 31.6). However, the time to colposcopy from the Pap test was also not significantly different between 2019 and 2020 (days; 27.9 ± 10.1 and 27.2 ± 18.1, *P* = .576) (Fig. 1). In addition, the Pap results and residence did not change the lead time in women in 2020 compared to 2019. In women with SCC, the lead time is longer in 2020 (days; 25.0 vs 14.0); however, this difference was not statistically significant due to the small population.

### 3.3 Results of the colposcopy

The colposcopy results are summarized in Table 2. More women in 2020 were diagnosed with high-grade dysplasia after colposcopy with punch biopsy (≥CIN2; 39.1% vs 34.7%, *P* = .124, ≥CIN3; 21.4% vs 13.1%, *P* < .001) and underwent excision procedures (29.5% vs. 25.4%, p = 0.118). The time to the excision procedure from the Pap test was similar between 2019 and 2020 (days, 59.9 ± 33.6 vs 54.8 ± 22.4, *P* = .114). The final results of the excision procedure were also worse in 2020 than in 2019 (≥CIN2; 87.4% vs 83.0%, *P* = .038, ≥CIN3; 65.5% vs 55.1%, *P* < .001). Resection margins involving high-grade dysplasia were detected more often in women in 2020; however, the difference was not significant (25.3% vs 20.1%, *P* = .279).

**Table 2:** Results of the colposcopy-directed biopsy.

	Before the COVID-19 era (2019, n = 621)	In the COVID-19 era (2020, n = 523)	*P*
Results of the colposcopy-directed biopsy			.047
Normal	312 (51.8)	260 (50.5)	
CIN1	81 (13.5)	54 (10.5)	
CIN2	130 (21.6)	91 (17.7)	
CIN3/CIS	76 (12.6)	106 (20.6)	
Invasive cancer	3 (0.5)	4 (0.8)	
Excision procedure			.118
No	453 (74.6)	360 (70.5)	
Yes	154 (25.4)	151 (29.5)	
Time to excision from the Pap	59.9 ± 33.6	54.8 ± 22.4	.114
Final pathological results of the excision procedure			.182
No residual lesion	16 (10.4)	14 (9.3)	
CIN1	10 (6.5)	5 (3.3)	
CIN2	43 (27.9)	33 (21.9)	
CIN3/CIS	80 (51.9)	95 (62.9)	
Invasive cancer	5 (3.2)	4 (2.6)	
Resection margin status			.279
Clear	123 (79.9)	112 (74.7)	
Involved by HSIL	31 (20.1)	38 (25.3)	

CIN = cervical intraepithelial neoplasia, CIS = carcinoma in situ, COVID-19 = Coronavirus disease, HSIL = high-grade squamous intraepithelial lesions, Pap = Papanicolaou test.

### 3.4 Risk factors for a long lead time (>6 weeks) to colposcopy from the Pap test

The patients’ residence (not Seoul; odds ratio [OR]=1.649, 95% confidence interval CI] = 1.180–2.305; *P* = 0.003), institution where the Pap test is performed (not same as those of colposcopy; OR = 2.218, 95% CI = 1.609–3.057; *P* < .001), and results of the Pap test (high-grade cytology ≥ASC-H; OR = 0.538, 95% CI = 0.351–0.825; *P* = 0.005) were associated with a long lead time to colposcopy of >6 weeks (Table 3). The age and year when women visited the institution did not affect the lead time (OR = 1.045, 95% CI = 0.767–1.423; *P* = .781).

**Table 3:** Adverse factors for a long lead time (>6 weeks) to colposcopy from the Pap.

	Univariate	*P*	Multivariate	*P*
Age				
>35	Reference			
≤35	0.946 (0.695–1.288)	.724		
Residence				
Seoul	Reference		Reference	
Others	1.657 (1.192–2.304)	.003	1.649 (1.180–2.305)	.003
Institution of the Pap				
Same institution	Reference		Reference	
Others	2.140 (1.511–2.838)	<.001	2.218 (1.609–3.057)	<.001
Results of the Pap				
<LSIL	Reference		Reference	
ASC-H<	0.637 (0.419–0.967)	.034	0.538 (0.351–0.825)	.005
Year				
2019	Reference			
2020	1.045 (0.767–1.423)	.781		

ASC-H = atypical squamous cells cannot exclude high-grade squamous intraepithelial lesions, LSIL = low-grade squamous intraepithelial lesions, Pap = Papanicolaou test.

## 4 Discussion

Our study showed that the lead time to colposcopy from the abnormal Pap test was not delayed in the COVID-19 era compared to the previous year. This result is consistent with the Pap results and the residence of patients. Contrastingly, women diagnosed with LSIL (24.3 vs 25.5 days), ASC-H (25.6 vs 29.2 days), and HSIL (23.2 vs 25.3 days) were followed up earlier in 2020 than 2019, although this difference is not statistically significant. In addition, women living in rural areas did not delay the follow-up visit to the institution in Seoul despite the COVID-19 pandemic (30.9 vs 32.0 days). However, regardless of whether the visit time was before or during the COVID-19 era, women not living in Seoul (OR = 1.649) or referred from other medical institutions (OR = 2.218) significantly delayed the follow-up visit by >6 weeks.

The colposcopy results were significantly different between 2019 and 2020 in this study. High-grade lesions were detected more frequently in women who underwent colposcopy in 2020, although the time to the excision procedure from the abnormal Pap test was not delayed (54.8% vs 59.9%). A selection bias could be a reason for this result. It is likely that all women with abnormal Pap results did not visit the institution for colposcopy because of an unusual situation due to COVID-19. The number of women who visited the institution for colposcopy in 2020 decreased to 84.2% (523/621) as of 2019. The physician performing the Pap test may provide counseling to minimize follow-up, or patients may skip the follow-up visit arbitrarily.

In a previous retrospective study, the median length of time from the abnormal Pap test to colposcopy was 99 days.^[8]^ Notably, older women with ASC-H results were more likely to delay colposcopy. However, there are some discrepancies in barriers to follow-up to abnormal Pap tests between reports.^[9,10]^ The authors stated that this delay may lead to a progression to invasive cancer in these high-risk women; thus, future research and guidelines must focus on these women to improve the adherence to follow-up procedures. Several countries released their recommendations about the timing of colposcopy after abnormal Pap tests.^[5]^ Notably, the United Kingdom, New Zealand, Canada, and Australia recommend immediate colposcopy within 2 weeks in women with SCC.^[11–14]^ In the case of high-grade cytological abnormalities, colposcopy within 2 weeks to 3 months is recommended. This clinical range is wider in the case of low-grade cytological abnormalities, with a maximum of 24 months in young women aged <25 years. Although colposcopy guidance and quality indicators from regional and national colposcopy societies exist, the recommendations are widely varied, especially considering age, HPV infection state, or persistent abnormal cytology.

This study first attempted to analyze the vague anxiety of medical access after abnormal screening for cervical cancer during the COVID-19 era and evaluate the barriers to the proper follow-up visit. However, this study has several limitations. First, selection bias may exist owing to the retrospective study design. The real population of missing women diagnosed with abnormal Pap results who were not followed up is unknown. In Korea, approximately 20,000 women with abnormal Pap in national cancer screening by the National Health Insurance Service (NHIS) results are reported annually.^[15]^ However, the exact ratio for follow-up after abnormal Pap results, including NHIS and private screening, has not been reported. Second, this study was conducted at a single institution. The situation of other tertiary or university hospitals was not reflected. Third, it was impossible to distinguish between involuntary delays, such as our institution's appointment system or the preferred physician's schedule.

## 5 Conclusions

Delays in the confirmatory testing after abnormal screening for cervical cancer due to the COVID-19 pandemic have not been identified in this study; however, delays by regional factors are revealed. A further nationwide analysis is needed to evaluate the rate of confirmatory tests after an abnormal Pap test to identify real missing cases at a high risk of cancer development, and we review the NHIS database based on the results of this study. A nationwide analysis can be helpful in improving the present referral process and establishing academic guidelines or regional policies for follow-up testing.

## Author contributions

**Conceptualization:** Miseon Kim, Bo Seong Yun.

**Data curation:** Miseon Kim.

**Formal analysis:** Miseon Kim.

**Supervision:** Bo Seong Yun.

**Writing – original draft:** Miseon Kim, Nara Lee, Bo Seong Yun.

**Writing – review & editing:** Miseon Kim, Nara Lee, Seyeon Won, Ju-Hyun Kim, Mi Kyoung Kim, Mi-La Kim, Yong Wook Jung, Bo Seong Yun, Seok Ju Seong.
